# Multidisciplinary Treatment: Follow-Up of Dental Autotransplantation for 10 Years

**DOI:** 10.1055/s-0043-1777048

**Published:** 2024-01-23

**Authors:** Elisa Souza Camargo, Rhafaela Ribeiro Silva, Ádelin Olívia Lopes Joly Rodrigues, Patricia Kern Di Scala Andreis, José Vinicius Bolognesi Maciel, Sônia Mara Luczyszyn, Evelise Machado de Souza, Everdan Carneiro, Nathália Juliana Vanzela, João Luiz Carlini

**Affiliations:** 1 Graduate Program in Dentistry – Orthodontics, School of Medicine and Life Sciences, Pontifícia Universidade Católica do Paraná, Curitiba, Paraná, Brazil; 2 Graduate Program in Dentistry – Restorative Dentistry, School of Medicine and Life Sciences, Pontifícia Universidade Católica do Paraná, Curitiba, Paraná, Brazil; 3 Graduate Program in Dentistry – Colletive Health, School of Medicine and Life Sciences, Pontifícia Universidade Católica do Paraná, Curitiba, Paraná, Brazi; 4 Undergraduate Program in Dentistry - Stomatology Department, Universidade Federal do Paraná, Curitiba, Paraná, Brazil; 5 Undergraduate Program in Dentistry - Periodontology, School of Medicine and Life Sciences, Pontifícia Universidade Católica do Paraná, Curitiba, Paraná, Brazil; 6 Graduate Program in Dentistry – Endodontics, School of Medicine and Life Sciences, Pontifícia Universidade Católica do Paraná, Curitiba, Paraná, Brazil; 7 Undergraduate Program in Dentistry – School of Medicine and Life Sciences, Pontifícia Universidade Católica do Paraná, Curitiba, Paraná, Brazil; 8 Undergraduate Program in Dentistry - Maxillofacial Surgery, Health Sciences Department, Universidade Federal do Paraná, Curitiba, Paraná, Brazil

**Keywords:** autotransplant, tooth avulsion, multidisciplinary care team

## Abstract

The objective is to present a clinical case of dental autotransplantation managed with surgery, orthodontics, endodontics, periodontics, and aesthetic rehabilitation. A 10-year-old boy sought treatment after avulsion of the maxillary left central incisor, which was not reimplanted. Based on anamnesis, clinical examination, and complementary examinations, agenesis of the maxillary and mandibular second premolars except the mandibular right second premolar was observed. After a multidisciplinary planning, the space in the maxillary left central incisor region was opened to receive the transplanted mandibular right second premolar. The receptor site was created in a single surgical procedure. Pulp necrosis was noted in the transplanted tooth, which was treated endodontically, and the agenesis spaces were closed using fixed orthodontic appliances. After removing the appliance, gingivectomy with osteotomy was performed in the maxillary right central incisor and the transplanted tooth regions to harmonize the height and shape of the gingival contour. Next, aesthetic readjustment was performed with tooth whitening, using office and home techniques, followed by microabrasion of the vestibular surface of the maxillary right central incisor. Direct composite resin restorations were placed in the maxillary incisors, and the teeth were rehabilitated using incisal and palatal guides. A multidisciplinary approach is essential for reestablishing the function and aesthetics of complex cases involving dental autotransplantation.

## Introduction

Autotransplantation stands out as a highly effective approach for restoring missing teeth, particularly when dealing with “immature” premolars. Research originating from the University of Oslo 1 has yielded compelling success rates, ranging from 75 to 91%. 2 The key to this success lies in meticulous patient selection and precise execution of surgical protocols. 2 This complex procedure involves transplanting a tooth before its root development is complete and preserving pulp revascularization and vitality. As a result, the transplanted tooth retains the potential to erupt and, notably, stimulate alveolar bone growth. 1 This underscores the clinical significance of autotransplantation in dental restoration.

Premolar teeth are frequently chosen for autotransplantation procedures, 3 especially in cases of dental crowding or dentoskeletal issues. A specific subset of premolar autotransplantations, directed at restoring missing anterior teeth, has demonstrated remarkable success rates and sustained positive clinical outcomes. 4 However, it is vital to recognize that achieving patient satisfaction in terms of smile aesthetics can be challenging due to the inherent variability in dental anatomy and the complex spatial arrangement of transplanted premolars within the maxillary central incisor region. 5 Therefore, the integration of a multidisciplinary treatment approach is essential to address these intricate clinical considerations. 6

Autotransplantation improves dental function and aesthetics. Success depends on periodontal ligament healing, regardless of tooth maturity. 7 Pulp healing is anticipated in developing teeth but not in mature ones. 8 Moreover, autotransplantation preserves bone tissue, showcasing its holistic dental care benefits. 9

This report highlights a clinical case involving autotransplantation of a mandibular premolar to replace a missing maxillary central incisor. The patient had additional missing teeth due to agenesis, and the treatment included surgery, orthodontics, endodontics, and aesthetic rehabilitation.

## Case Report

**Fig. 1:**
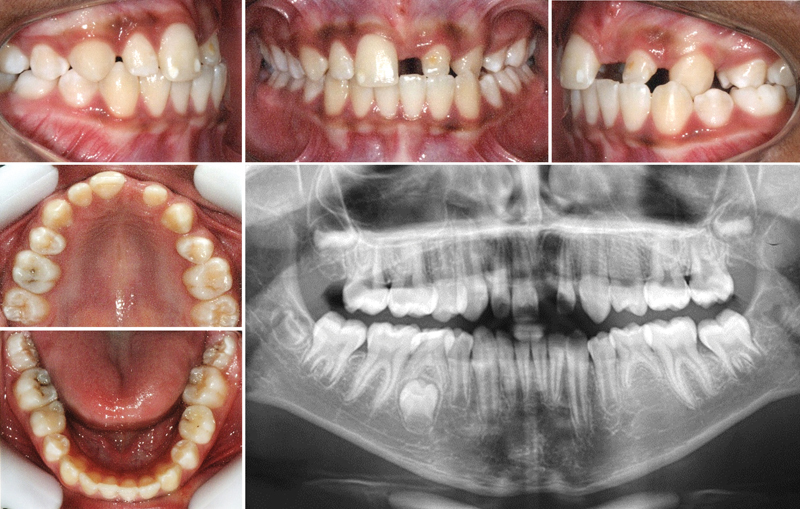
Initial intraoral photographs and panoramic radiograph (of a 11.2-year-old) with avulsion of the maxillary left central incisor and agenesis of the maxillary and mandibular premolars except the mandibular right second premolar.

A 10-year-old boy presented with complaint of loss of the maxillary left central incisor due to trauma and avulsion, and the consequent lack of function and unsatisfactory aesthetics in the region. Initial intraoral examination revealed a mixed dentition, presence of deciduous mandibular second molars without mobility, absence of the maxillary second premolars and maxillary left central incisor, and a discrepancy between the mesiodistal diameters of the maxillary lateral incisors. The molar relationship was class II bilaterally, with unilateral left anterior and posterior crossbite. Panoramic radiograph showed agenesis of all the second premolars except the mandibular right one, which had a fully formed crown ( Fig. 1 ).

Considering treatment options such as prosthetic rehabilitation with an implant-supported fixed prosthesis and autotransplantation, we decided to autotransplant the mandibular right second premolar to the avulsed maxillary left central incisor region and close the second premolar spaces caused by agenesis and autotransplantation.

Favorable conditions such as appropriate age of the patient, receptor site, and donor tooth (mandibular right second premolar in stage 7 of root development 10 ) for autotransplantation in the region of the avulsed tooth were observed.

### Orthodontics

Orthodontic treatment began with rapid maxillary expansion using a hyrax-type palatal expansion appliance to increase the space in the maxillary left central incisor region for receiving the donor tooth and to correct the crossbite. An edgewise fixed appliance with brackets having a 0.022 inch × 0.028 inch slot (American Orthodontics, Sheboygan, WI, United States) was used in both dental arches for tooth alignment and leveling. Additional space opening with the aid of a nickel-titanium open coil spring between the maxillary right central incisor and maxillary left lateral incisor was performed. Autotransplantation was accomplished at this stage, when the premolar root had completed two-thirds of its formation, considered the best time for this procedure. 11

Space closure in missing second premolars and donor regions was performed; anchorage from mini-implants and Bull loops with helicoids on a stainless steel 0.019 inch × 0.025 inch rectangular archwire (American Orthodontics) assisted mesialization of the mandibular permanent molars.

**Fig. 2:**
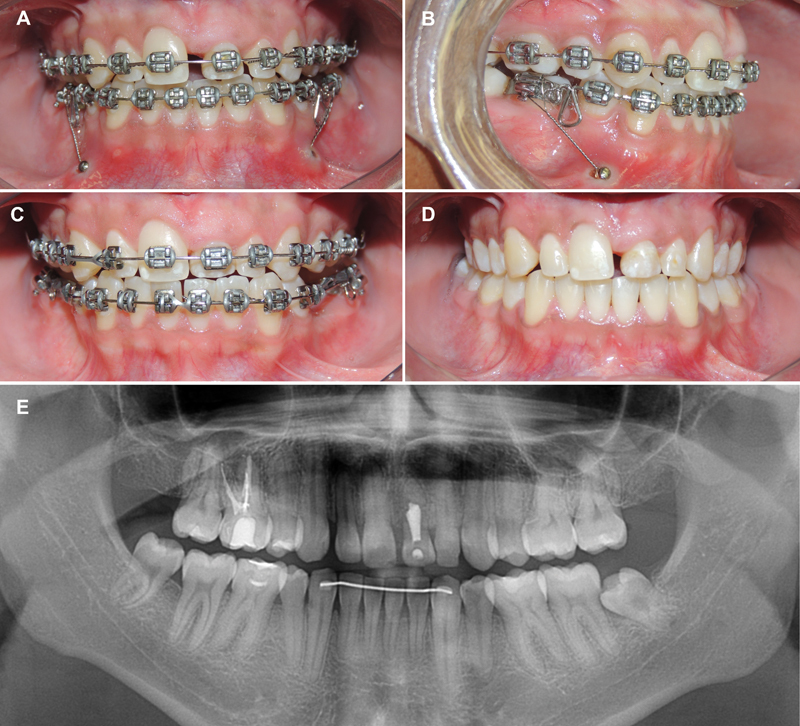
( **A** ) Space closure with Bull loops and anchorage from mini-implants. ( **B** ) Closed spaces. ( **C,D** ) Completion of orthodontic treatment (18.6-year-old). ( **E** ) Final panoramic radiograph.

The transplanted tooth was included in orthodontic treatment at the finishing stage to minimize the risk of root resorption. At the end of the orthodontic treatment, the fixed appliance was removed, and a canine-to-canine bonded fixed retainer in the mandibular arch and a wraparound-type removable appliance in the maxillary arch were installed ( Fig. 2 ).

### Surgery

The surgical procedure of autotransplantation was performed using an implant conical drill (BLT System, Straumann, Basel, Switzerland) sequence and abundant irrigation in the maxillary left central incisor region. Milling was started with a ø 2.0-mm drill for setting the drill direction, ensuring to not invade the cortical bone plates that separate the adjacent teeth. Next, ø 2.8-, ø 3.5-, and ø 4.2-mm drills of the same system were used to enlarge the osteotomy site, avoiding trauma to the bone tissue. Minor adjustments required to adapt the transplanted tooth were performed using a spherical bur (no. 8). 12

**Fig. 3:**
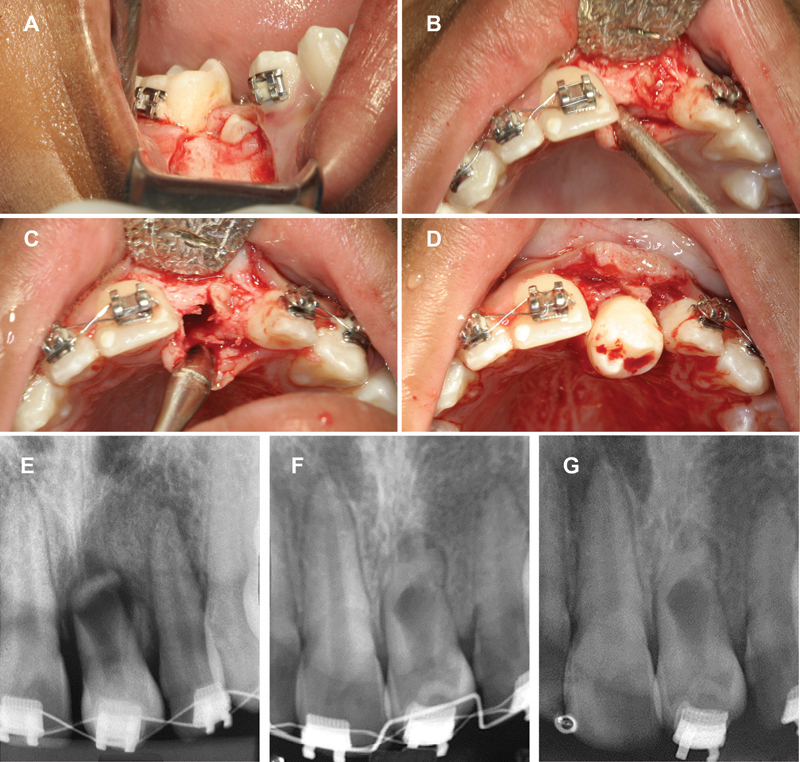
( **A** ) Right mandibular second premolar extraction. ( **B** ) Raised gingival flap. ( **C** ) Exposure of bone opening at the receptor site. ( **D** ) Transplanted tooth to the maxillary left central incisor region. ( **E** ) Periapical radiography 10 days after surgery. ( **F** ) Periapical radiography control 1 year after surgery. ( **G** ) Periapical radiography at orthodontic finishing stage.

In the second surgical stage, the deciduous mandibular right second molar and mandibular right second premolar were extracted using the atraumatic tooth extraction kit (Thimon, São Paulo, SP, Brazil). The premolar was immediately transplanted into the alveolus of the maxillary left central incisor ( Fig. 3 ), positioned in infraocclusion, avoiding contact with the antagonist, 13 and immobilized with 3–0 silk sutures.

Ten days postoperatively, the sutures were removed, and a space maintainer fabricated using a 0.020-inch stainless steel wire was bonded to the buccal surfaces of the mandibular right first premolar and first molar to prevent tipping of the teeth until the orthodontic appliance was bonded.

### Endodontics

**Fig. 4:**
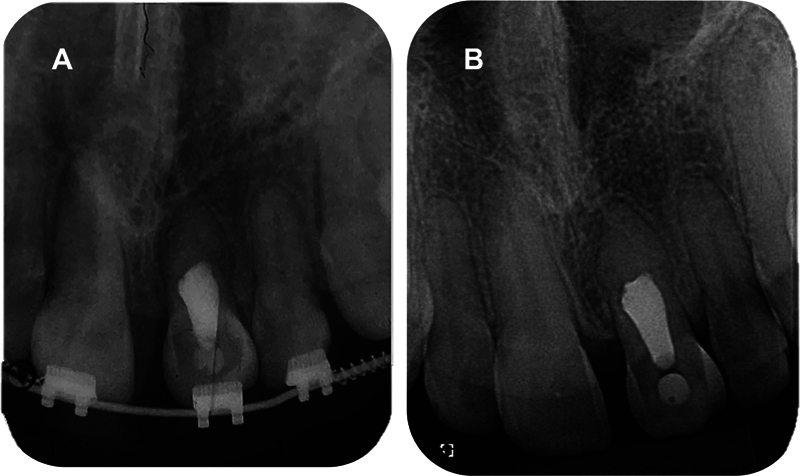
Follow-up periapical radiographies of the endodontic treatment. ( **A** ) Initial and ( **B** ) final.

Forty-five days after surgery, a cone beam computed tomography of the region with the transplanted tooth was performed for diagnosis. During the regular monthly visits, the tooth remained asymptomatic, and control periapical radiographs showed that a barrier of mineralized tissue had begun to form in the periapical region. After 12 months of clinical control, a fistula formed in the transplanted tooth, indicating pulpal necrosis. The pulp necrosis diagnosis was determined based on clinical and radiographic examination according to the American Association of Endodontists (AAE) guidelines. 14 The root canal was immediately accessed, irrigated with 2.5% sodium hypochlorite (NaOCl), and prepared with hand files, k-file sizes 90 to 140 (Dentsply Maillefer, Ballaigues, Switzerland) and an intracanal dressing of calcium hydroxide paste was placed (Ultradent Products, Inc., South Jordan, UT, United States). After 21 days, the intracanal dressing was removed, and the root canal was irrigated using ethylenediaminetetraacetic acid (EDTA) and 2.5% NaOCl, dried with paper cones, and mineral trioxide aggregate (MTA; Angelus, Londrina, PR, Brazil) plug was performed to permit root canal filling. The rest of the canal was obturated with gutta-percha using a thermoplastic technique and a root canal sealer (AH Plus; Dentsply Maillefer). The pulp chamber was sealed with glass ionomer cement ( Fig. 4 ).

### Aesthetic Rehabilitation

**Fig. 5:**
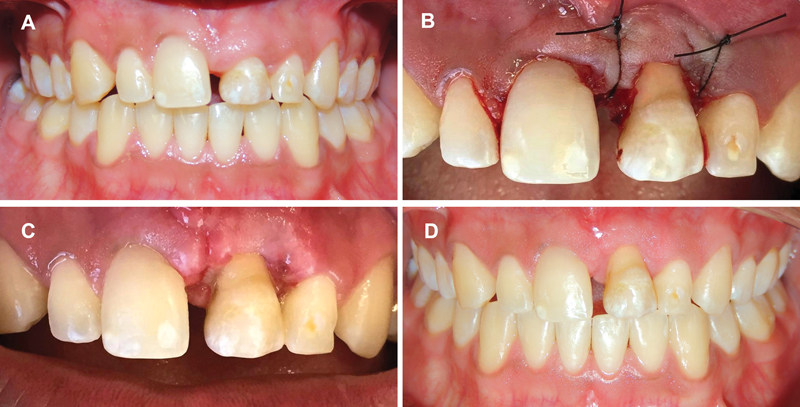
( **A** ) Discrepancy in the height and shape of the gingival contour after dental autotransplantation. ( **B** ) Immediate postoperative period after gingivectomy with osteotomy on the maxillary right central incisor and transplanted tooth. ( **C** ) Postoperative healing after 7 days. ( **D** ) Follow-up of 3 months after gingival surgery.

The discrepancy in the height and shape of gingival contour was corrected by gingivectomy with osteotomy on the transplanted tooth that would be rehabilitated as the maxillary left central incisor ( Fig. 5 ).

Considering the difference in color saturation among teeth, tooth whitening with 35% hydrogen peroxide (Whiteness HP® FGM Dent Scare, Joinville, SC, Brazil), and supervised homemade with 10% carbamide peroxide (Whiteness Perfect 10%; FGM Dent Scare) combining office and home techniques was performed. After ensuring color stability and hydration of the teeth, and microabrasion with 6% hydrochloric acid associated with silicone carbide particles (Whiteness RM; FGM Dent Scare) in regions with enamel hypoplasia, a desensitizing gel was applied (Desensibilize KF; FGM Dent Scare), and the enamel surface was polished, reducing evidence of white spots.

After 2 weeks, the diastemas between the incisors were closed, re-anatomization was performed, and dental proportions were corrected. Restorations were performed individually, using incremental and layered techniques, and finished by polishing.

The restorative process was initiated by etching of teeth with 35% phosphoric acid (Ultra-Etch, Ultradent Products, Inc.) using a universal adhesive system (Ambar Universal Adhesive APS; FGM Dent Scare). Enamel shade A1 and dentin shade DA1 (Opallis; FGM Dent Scare) microhybrid resins were used. To veneer the transplanted tooth and modify its anatomy, a DB1 dentin microhybrid resin with the highest color value was applied to the entire buccal surface, further whitening the substrate to bring harmony with the adjacent teeth.

**Fig. 6:**
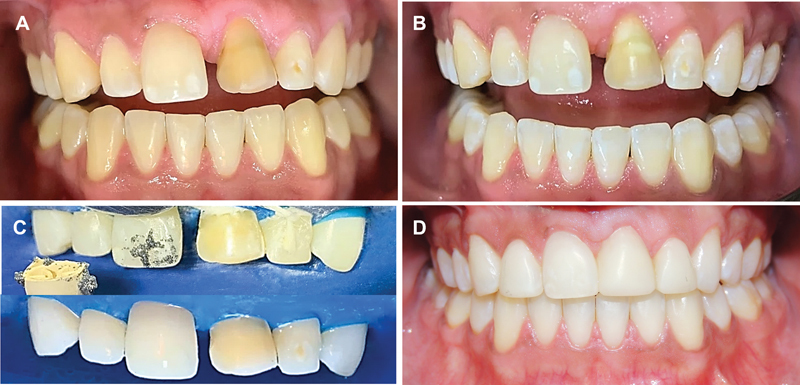
( **A** ) Initiation of tooth whitening. ( **B** ) Result after first in-office whitening session. ( **C** ) Microabrasion. ( **D** ) Aesthetic restorations with direct composite resin technique using incisal and palatal guides on maxillary central and lateral incisors and result after aesthetic finishing.

Subsequently, occlusal adjustments and finishing were performed with diamond tips (KG Sorensen, Barueri, SP, Brazil), sandpaper disks (Sof-Lex Pop On; 3M ESPE, São Paul, SP, Brazil), and sandpaper strips (Epitex; GC South American) for the interproximal areas. For polishing and enhancing final brightness, abrasive pads (Optimize, TDV Dental, Pomerode, SC, Brazil) and polishing paste (Diamond Excel, FGM Dent Scare) with felt-buffing disk were used to achieve the final gloss ( Fig. 6 ).

**Fig. 7:**
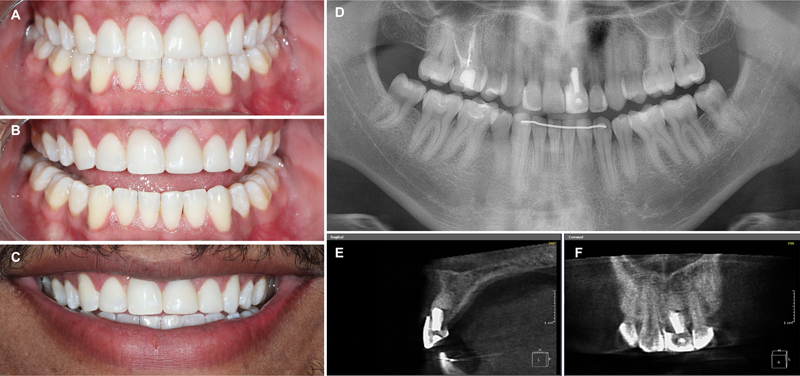
Follow-up 10 years after dental transplantation. ( **A–C** ) Intra- and extraoral images show healthy supporting tissues and good smile aesthetics. ( **D** ) Panoramic radiograph control. ( **E,F** ) The tomographic image suggests a good outcome of the transplanted tooth.

After the aesthetic rehabilitation, a new maxillary removable orthodontic retention appliance was manufactured. At the end of the treatment, 8 years after autotransplantation, the transplanted tooth had a good outcome, with healthy supporting tissues and excellent aesthetic and functional results ( Fig. 6 ), which were maintained up to 10 years of follow-up after dental transplantation ( Fig. 7 ). The patient was satisfied with the results of the multidisciplinary treatment.

## Discussion

This case presents the successful autotransplantation of a mandibular premolar into the maxilla as a replacement for the left central incisor in a 12-year-old boy. Avulsion of the anterior teeth is more prevalent in children aged 9 to 12 years, especially maxillary central incisors, 15 and different types of treatment are possible. In patients in the age range of the patient in the reported clinical case, insertion of dental implants and prostheses was not the best treatment option, and autotransplantation was considered the right option. Moreover, the agenesis of three premolars and the fourth one being in the developmental phase suitable for autotransplantation reinforced the choice of the proposed treatment. We waited until two-thirds of the root of the mandibular right second premolar was formed before performing autotransplantation, as the prognosis is more favorable in this phase. 6 11

Regarding the surgical procedure, extreme care was taken to ensure that the tissues adjacent to the transplanted tooth were intact, to enable continued root formation and regeneration of the periodontal ligament in the new recipient site. 13 For better stability and prognosis, the autotransplanted tooth was kept in disocclusion, as occlusal interference could affect apexification and pulp vitality. 13 This case was successful because we respected the main predictors of success, such as the short extraoral time of the tooth during transplantation, minimal damage to the root surface, maintenance of the quality of the surrounding tissues, and maintaining adequate immediate postoperative oral hygiene. 16

Progressive pulp obliteration is common after autotransplantation. 17 Despite following all protocols, the transplanted tooth developed pulp necrosis within a year due to dental trauma or bacterial contamination. To address this, endodontic treatment was conducted using MTA for its suitable properties and biocompatibility. 18

Orthodontic treatment was important to correct the crossbite, provide space for the transplanted tooth, and close the spaces caused by premolar agenesis and removal of the donor tooth, thereby achieving good occlusion. In the cases with active craniofacial growth, as in the patient in question, space closure is a reliable therapy 19 because prosthetic rehabilitation on dental implants at an early age leads to infraocclusion of the rehabilitated area. 20

To enhance aesthetic rehabilitation, a gingivectomy with osteotomy was performed in the transplanted maxillary central incisor area to reestablish the biological width and improve smile aesthetics. 21 After healing, plaster models of both dental arches were used for a diagnostic wax-up and guides. This ensured a harmonious dental composition, achieved a proper golden proportion of teeth, 22 and determined the necessary grinding for restoration space, particularly in the buccal and palatal areas of the transplanted tooth.

In this clinical case, enamel whitening was performed using office and home techniques to whiten and level the color of dental substrates. 23 The treatment performed in the office allowed selective whitening of the teeth with high chroma, which, when complemented with the home technique, provided greater color balance of the substrate of the set, in addition to providing greater control of dental sensitivity.

With the improved color of the dental substrate obtained by bleaching, minimally invasive, and selective wear, the dental structure was possible and composite resin was the material of choice. 24 This material has good mechanical strength, fluorescence similar to the dental structures, and adequate brightness when used for aesthetic rehabilitation in the anterior teeth, leading to longevity. 25 Moreover, it can reproduce the anatomy and appropriate dimensions of the teeth, which help preserve the integrity of periodontal health, provide satisfactory results to the patient, and balance aesthetics and health.

## Conclusion

This clinical case involved the treatment of tooth agenesis and avulsion of the maxillary central incisor with dental autotransplantation. Multidisciplinary treatment is essential to restore function and aesthetics in complex cases involving dental autotransplantation with excellence in its outcome.

## References

[1] Slagsvold O Bjercke B Applicability of autotransplantation in cases of missing upper anterior teeth Am J Orthod 1978 74 04 410 421 281144 10.1016/0002-9416(78)90063-5

[2] Machado L A do Nascimento R R Ferreira D M Mattos C T Vilella O V Long-term prognosis of tooth autotransplantation: a systematic review and meta-analysis Int J Oral Maxillofac Implants 2016 45 05 610 617 10.1016/j.ijom.2015.11.010 26696138

[3] Travess H Roberts-Harry D Sandy J Orthodontics. Part 8: extractions in orthodontics Br Dent J 2004 196 04 195 203 15039723 10.1038/sj.bdj.4810979

[4] Kim S Shin S J Park J W Long-term stability of autotransplanted premolars as a substitute for molars in adults J Endod 2016 42 08 1286 1290 27374818 10.1016/j.joen.2016.05.017

[5] Czochrowska E M Stenvik A Zachrisson B U The esthetic outcome of autotransplanted premolars replacing maxillary incisors Dent Traumatol 2002 18 05 237 245 12427198 10.1034/j.1600-9657.2002.00094.x

[6] Mendoza-Mendoza A Solano-Reina E Iglesias-Linares A Garcia-Godoy F Abalos C Retrospective long-term evaluation of autotransplantation of premolars to the central incisor region Int Endod J 2012 45 01 88 97 21906087 10.1111/j.1365-2591.2011.01951.x

[7] Jakse N Ruckenstuhl M Rugani P Kirnbauer B Sokolowski A Ebeleseder K Influence of extraoral apicoectomy on revascularization of an autotransplanted tooth: a case report J Endod 2018 44 08 1298 1302 29935869 10.1016/j.joen.2018.04.016

[8] Tsukiboshi M Autotransplantation of teeth: requirements for predictable success Dent Traumatol 2002 18 04 157 180 12442825 10.1034/j.1600-9657.2002.00118.x

[9] Velozo C Nogueira L R Nogueira Filho L R Capistrano A de Albuquerque D S Tooth autotransplantation using an interdisciplinary approach to rehabilitation in a young patient: case report with 7-year follow-up Dent Traumatol 2021 37 03 521 530 33269534 10.1111/edt.12637

[10] Nolla C M The development of permanent teeth ASDC J Dent Child 1960 27 253 266

[11] Freitas R J Dias L S Aiello C A Reabilitação dentária anterior com a utilização de transplante dentário autógeno de dente posterior: relato de caso Rev Odontol UNESP 2013 41 162

[12] Medeiros P J Cirurgia dos dentes inclusos: extração e aproveitamentoIn: Cirurgia dos dentes inclusos: extração e aproveitamento2003 147

[13] Park S Y Choi S C Choi B J Kim S J Park J H The autotransplantation and orthodontic treatment of multiple congenitally missing and impacted teeth J Clin Pediatr Dent 2012 36 04 329 334 23019827 10.17796/jcpd.36.4.n3013h2j15v35030

[14] AAE Glossary of Endodontic Terms 10th ed. Chicago, IL American Association of Endodontists 2020

[15] Glendor U Epidemiology of traumatic dental injuries: a 12 year review of the literature Dent Traumatol 2008 24 06 603 611 19021651 10.1111/j.1600-9657.2008.00696.x

[16] Grisar K Smeets M Ezeldeen M Survival and success of autotransplanted impacted maxillary canines during short-term follow-up: a prospective case-control study Orthod Craniofac Res 2021 24 02 222 232 32777135 10.1111/ocr.12422

[17] Czochrowska E M Stenvik A Album B Zachrisson B U Autotransplantation of premolars to replace maxillary incisors: a comparison with natural incisors Am J Orthod Dentofacial Orthop 2000 118 06 592 600 11113791 10.1067/mod.2000.110521

[18] Menezes R da Silva Neto U X Carneiro E Letra A Bramante C M Bernadinelli N MTA repair of a supracrestal perforation: a case report J Endod 2005 31 03 212 214 15735473 10.1097/01.don.0000137639.85637.67

[19] Murri Dello Diago A Apponi R Colombini V Mordini L Ideo F Complex implant-prosthetic rehabilitation following sports trauma with 14 years of follow-up: case report Dent J (Basel) 2021 9 01 6 33435430 10.3390/dj9010006 PMC7827219

[20] Kamatham R Avisa P Vinnakota D N Nuvvula S Adverse effects of implants in children and adolescents: a systematic review J Clin Pediatr Dent 2019 43 02 69 77 30730793 10.17796/1053-4625-43.2.1

[21] Lanning S K Waldrop T C Gunsolley J C Maynard J G Surgical crown lengthening: evaluation of the biological width J Periodontol 2003 74 04 468 474 12747451 10.1902/jop.2003.74.4.468

[22] Levin E I Dental esthetics and the golden proportion J Prosthet Dent 1978 40 03 244 252 279670 10.1016/0022-3913(78)90028-8

[23] Goettems M L Fernandez M DS Donassollo T A Henn Donassollo S Demarco F F Impact of tooth bleaching on oral health-related quality of life in adults: a triple-blind randomised clinical trial J Dent 2021 105 103564 33359042 10.1016/j.jdent.2020.103564

[24] Comba A Vergano E A Baldi A 5-year retrospective evaluation of direct composite restorations in orthodontically treated patients J Dent 2021 104 103510 33130052 10.1016/j.jdent.2020.103510

[25] Ferracane J L Resin composite: state of the art Dent Mater 2011 27 01 29 38 21093034 10.1016/j.dental.2010.10.020

